# Supplementary material for: Effects of extreme meteorological factors and high air pollutant concentrations on the incidence of hand, foot and mouth disease in Jining, China
Source: PeerJ. 2024 May 15;12:e17163. doi: 10.7717/peerj.17163 (PMC11102053; doi:10.7717/peerj.17163)
Supplement: Supplemental Information 3 [file peerj-12-17163-s003.pdf]

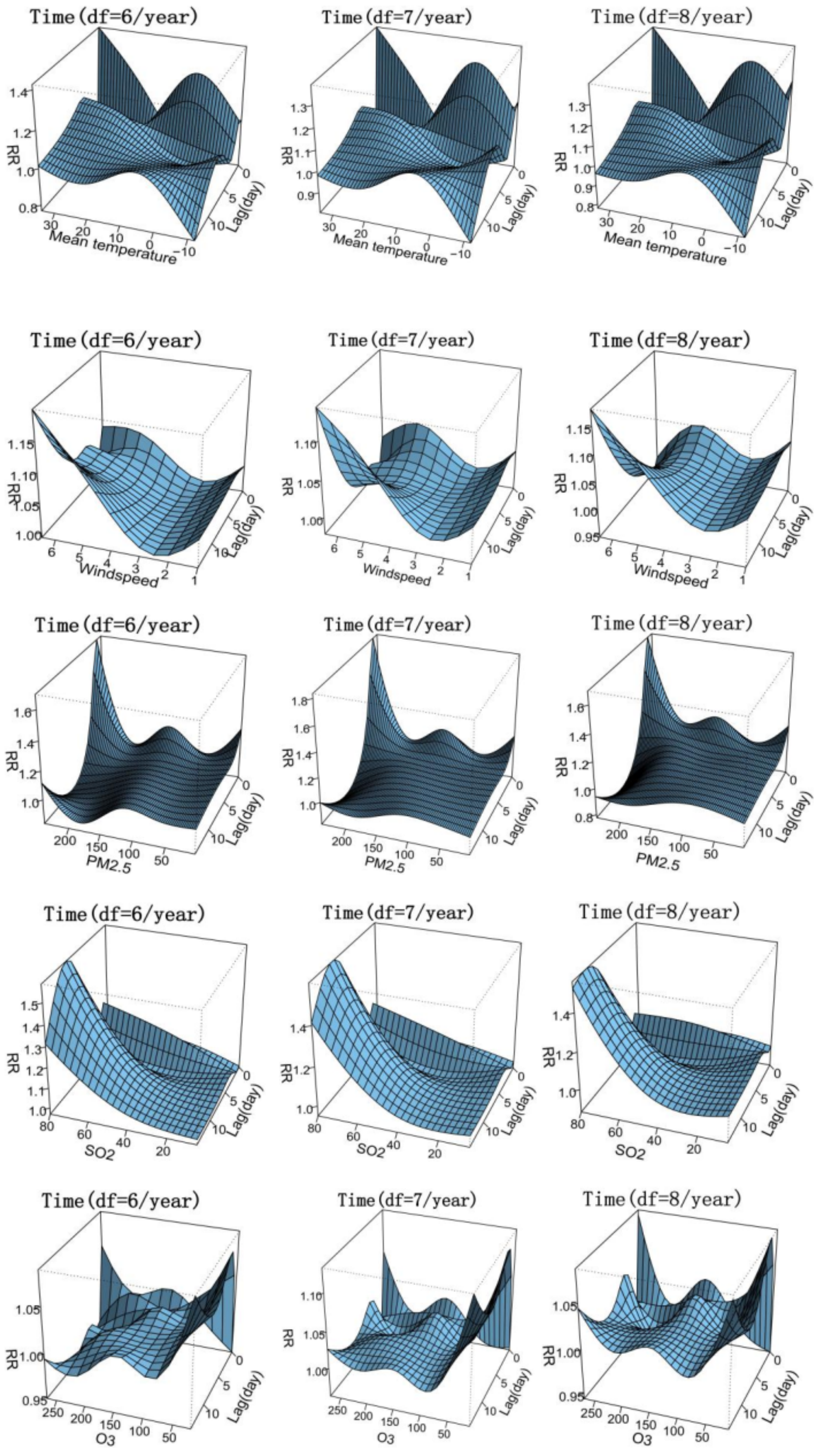

Fig.S1 Sensitivity analysis results of meteorological factors and air pollutions after changing the degree of freedom of time term

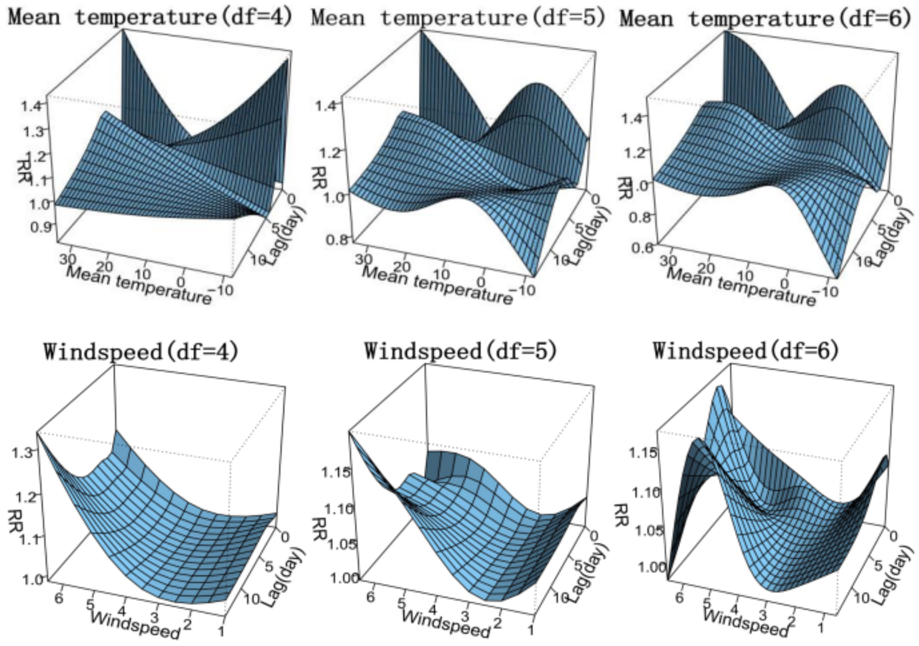

Fig.S2 Sensitivity analysis results of air pollution after changing the degree of freedom of time term

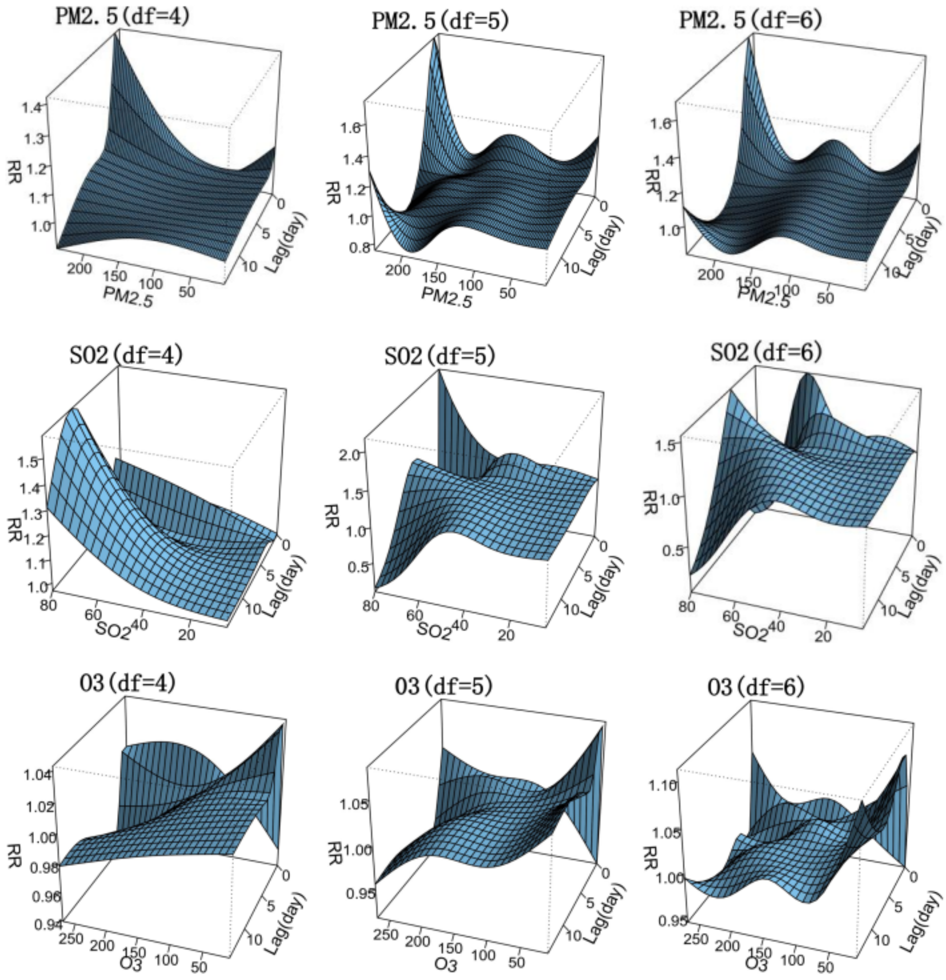

Fig.S3 Sensitivity analysis results after changing the degree of freedom of each air pollution
